# Supplementary figures and images for: Light affects behavioral despair involving the clock gene Period 1
Source: PLoS Genet. 2021 Jul 8;17(7):e1009625. doi: 10.1371/journal.pgen.1009625 (PMC8266116; doi:10.1371/journal.pgen.1009625)

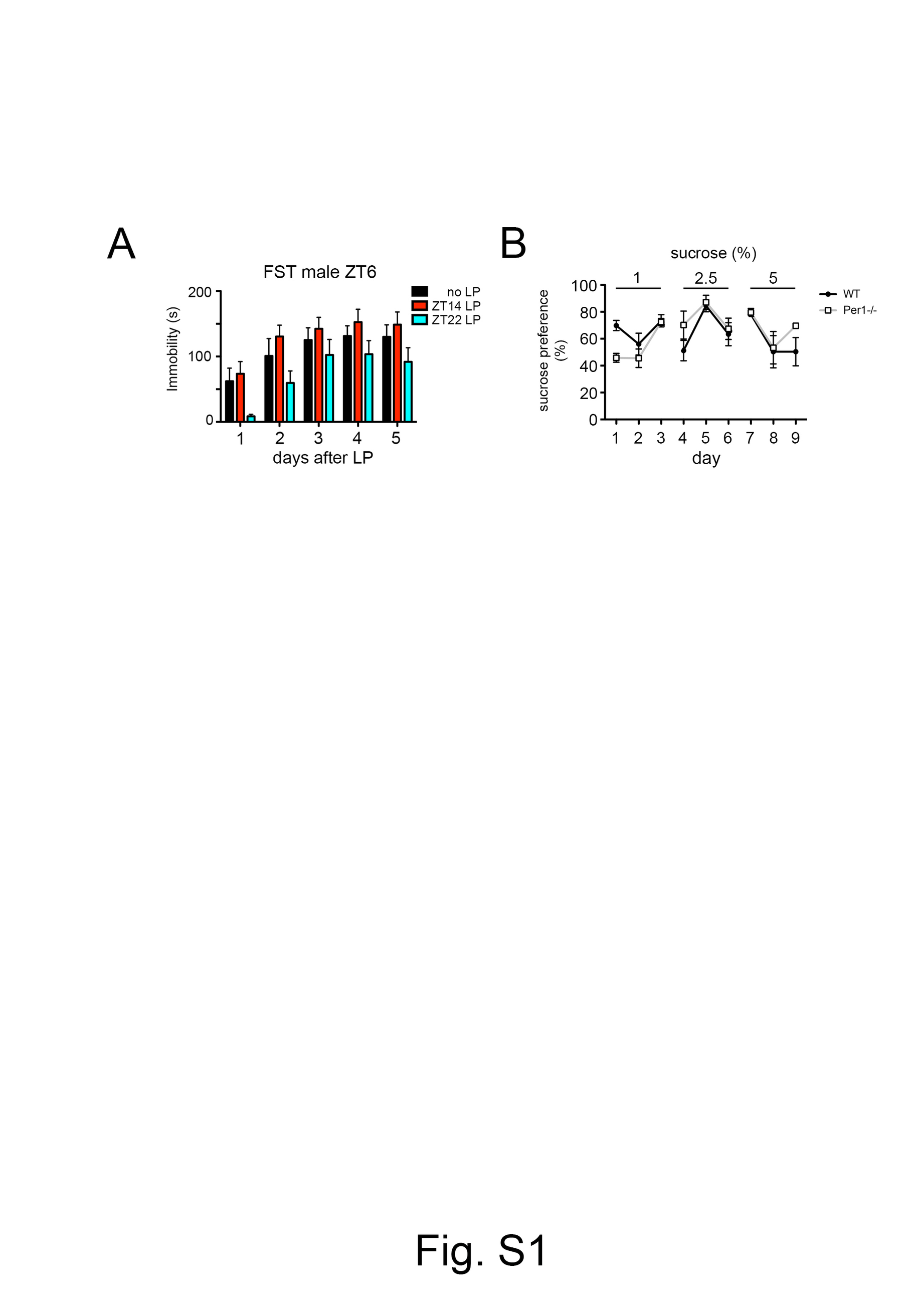

Supplement: S1 Fig — (A) Immobility time in the forced swim test (FST) of wild type male mice assessed over several days at ZT6 after no light pulse (LP) (black bars), after a LP at ZT14 (red bars), and after a LP at ZT22 (blue bars). Two-way repeated measures ANOVA (n = 13–15), ZT14 LP p = 0.44, ZT22 LP p = 0.14, values are means ± SEM. (B) Sucrose preference was tested allowing mice to choose between water or sucrose (1–5% (weight/vol)) over 3 days for each sucrose concentration. Two-way RM ANOVA revealed no differences between wild type (WT, n = 21, black lines) and Per1-/- mice (n = 18, grey lines), p = 0.67, values are means ± SEM. (TIF) [file pgen.1009625.s001.tif]

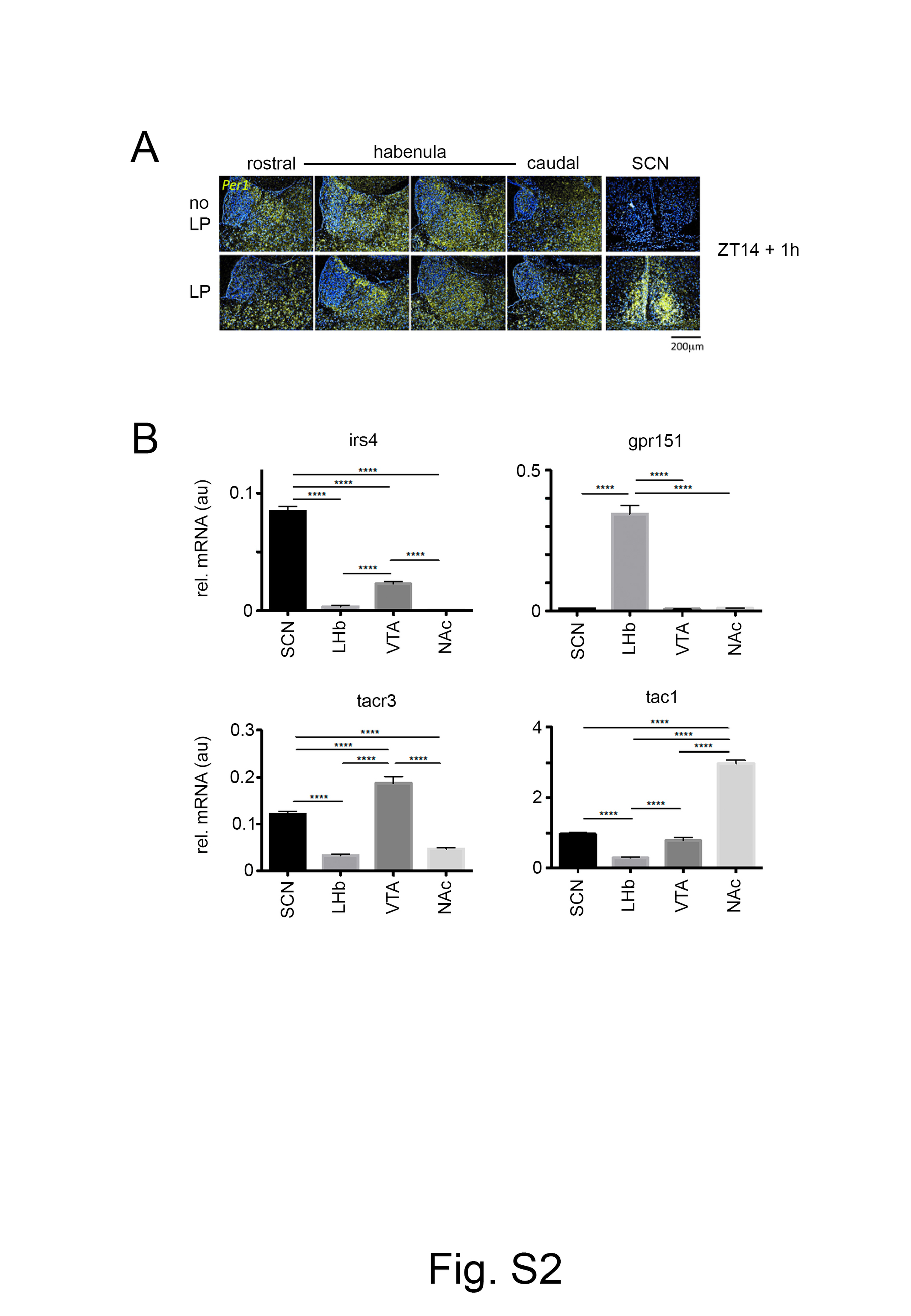

Supplement: S2 Fig — (A) Light induction of Per1 at ZT14 in the LHb and the SCN. Dark-field images of coronal sections containing the habenula (rostral to caudal, left panels) and the SCN as positive control (right panels). The yellow signal represents the hybridization signal detecting Per1 mRNA and blue represents Hoechst-dye stained cell nuclei. The MHb can be distinguished from the LHb by the densely packed blue-colored nuclei. Brain section of mice sacrificed 60 min. after the light pulse (bottom row) and the corresponding controls are shown (top row). Scale bar: 200μm. (B) Brain region specific markers for verification of isolated brain regions used for further analysis. Quantitative PCR comparing various genes in the SCN, LHb, VTA and NAc. The most specific gene for the SCN is irs4, for the LHb it is gpr151, for the VTA it is tacr3 and for the NAc it is tac1. One-way ANOVA with Tukey’s multiple comparisons test was used, n = 12–16, ****p<0.0001, values are means ± SEM. (TIF) [file pgen.1009625.s002.tif]

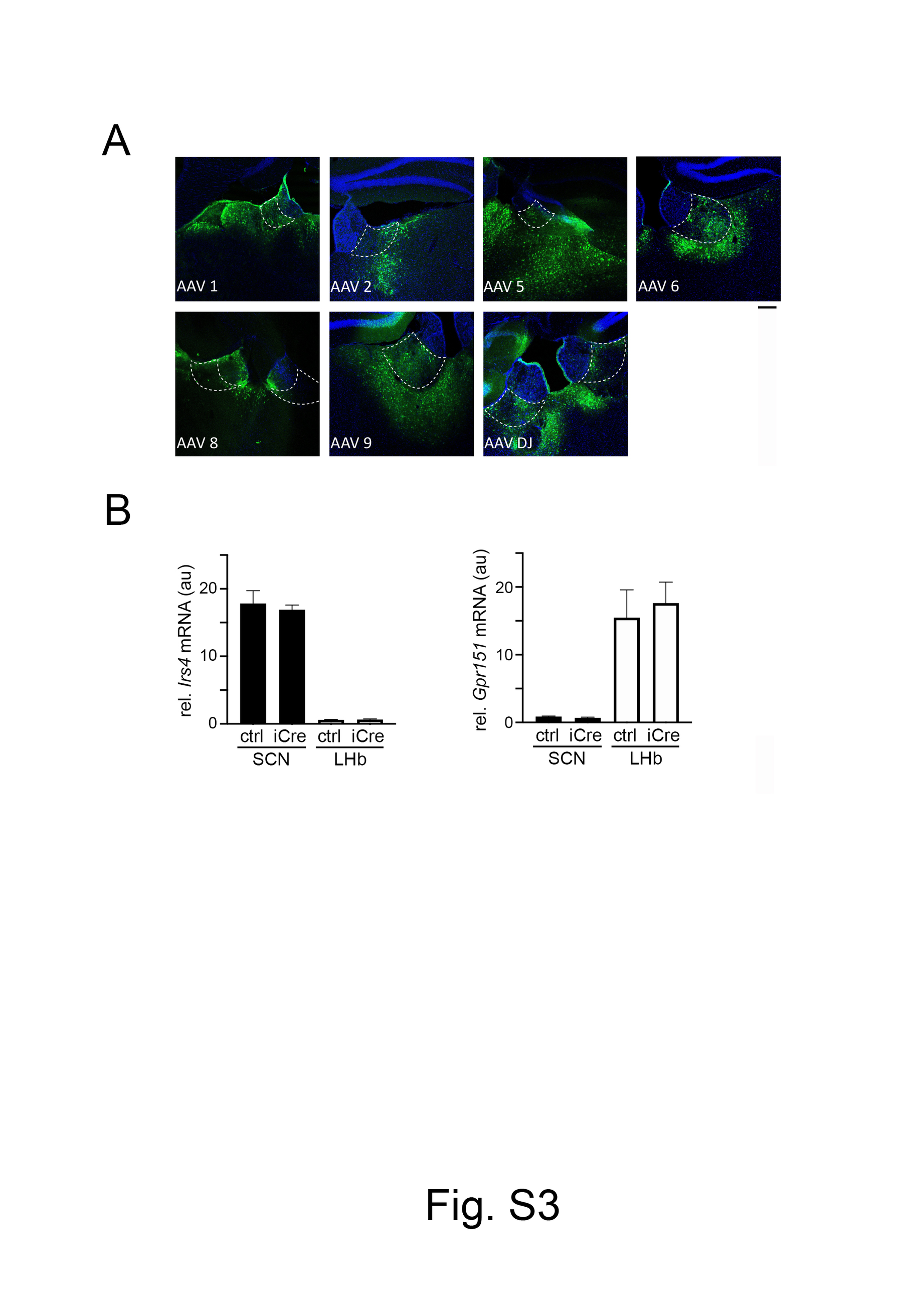

Supplement: S3 Fig — (A) Brain sections of the habenular region are shown. Optimization of infection efficiency was performed by testing different variants of adeno-associated virus (AAV) expressing GFP (green color). Blue indicates cell nuclei (DAPI staining). For the lateral habenula (hatched white lines) AAV6 appeared to have the most localized and strongest infection potential. Scale bar: 200 μm. (B) Tissue controls for PCR deletion verification of Per1 in Fig 3C. The Irs4 mRNA is only detected in SCN tissue and not the LHb (left panel). The Gpr151 mRNA is only detected in LHb tissue and not the SCN (right panel). (TIF) [file pgen.1009625.s003.tif]

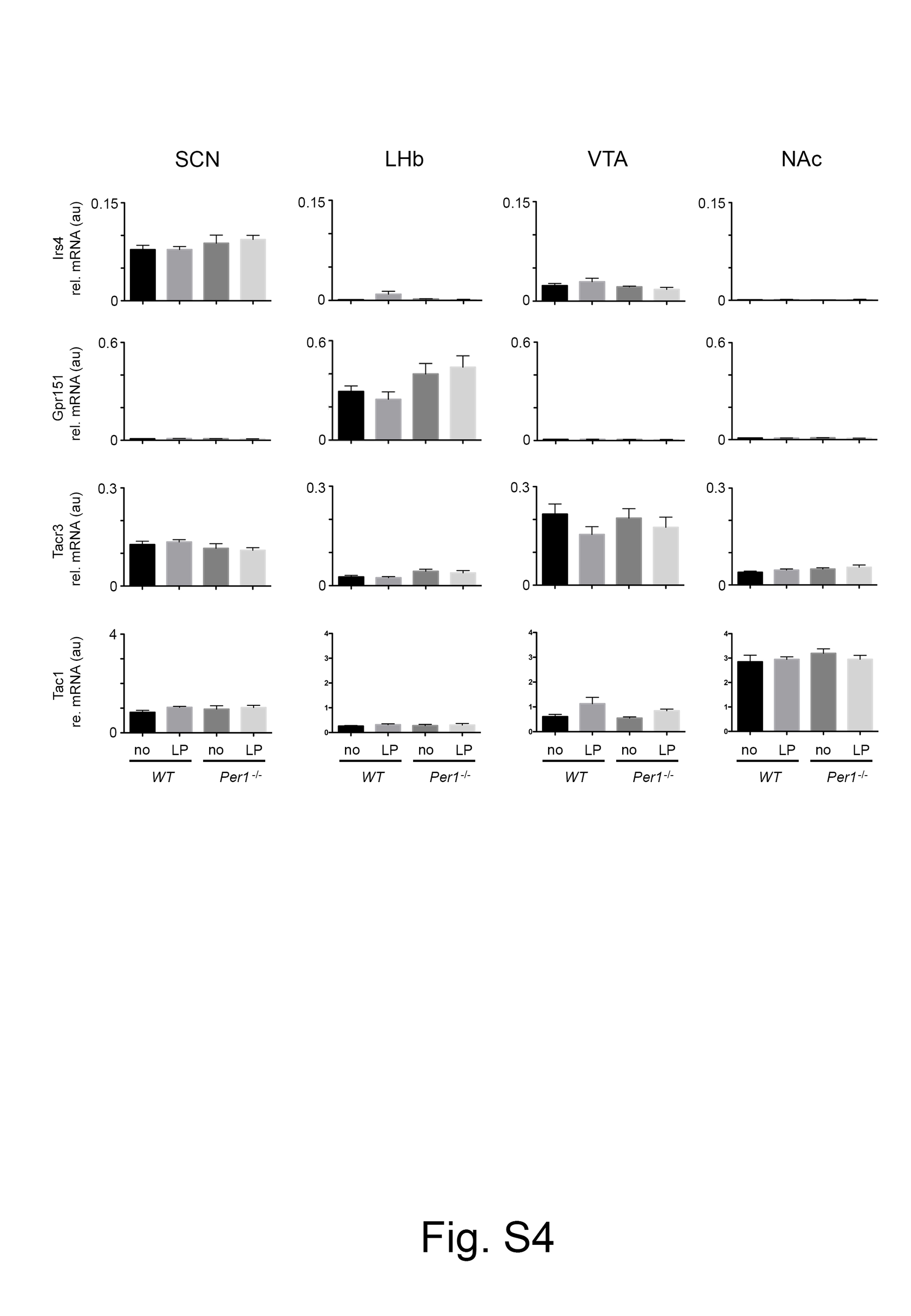

Supplement: S4 Fig — Quantitative PCR comparing various genes in the SCN, LHb, VTA and NAc. The most specific gene for the SCN is irs4, for the LHb it is gpr151, for the VTA it is tacr3 and for the NAc it is tac1. (TIF) [file pgen.1009625.s004.tif]

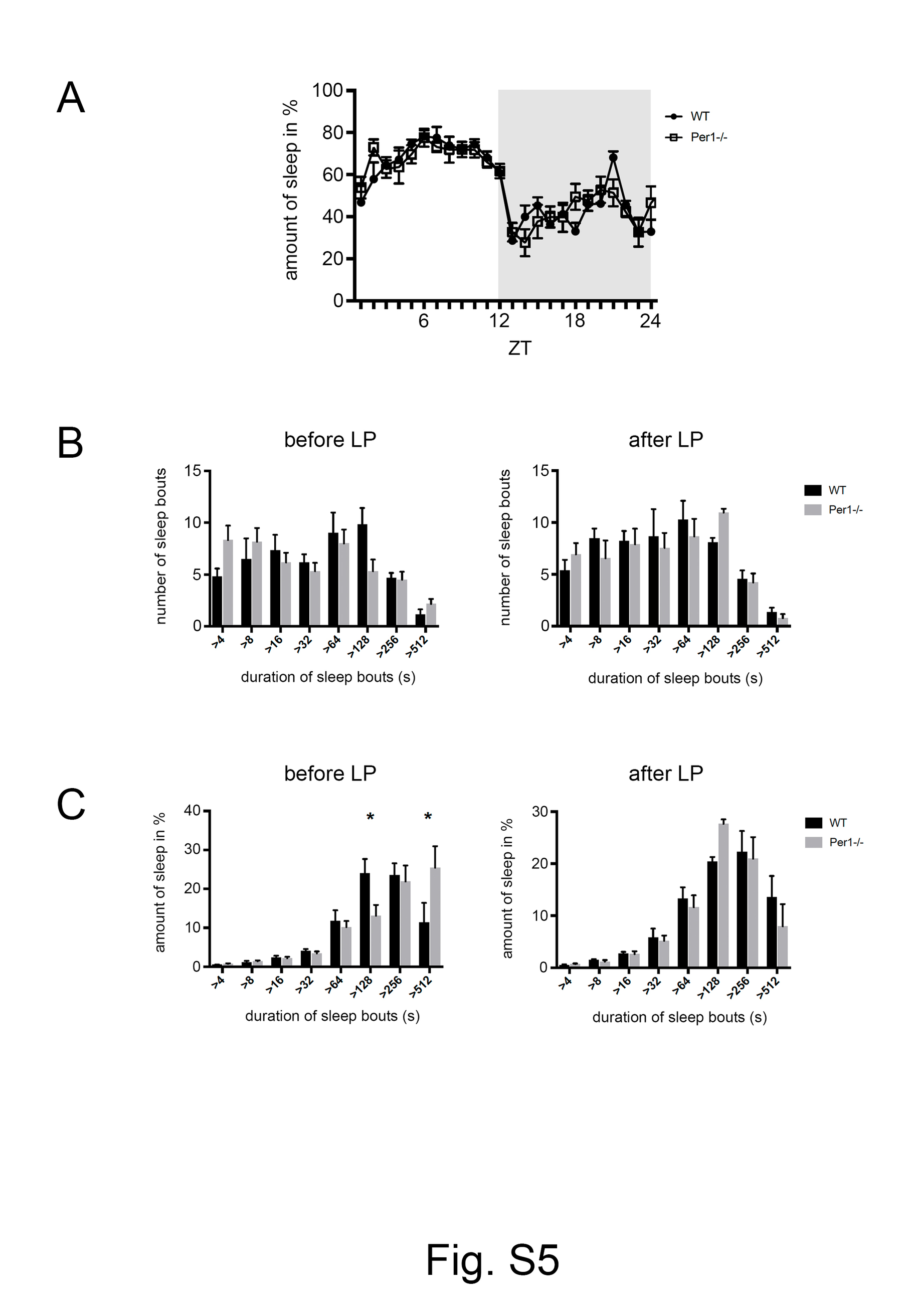

Supplement: S5 Fig — (A) Amount of sleep in % over 24 hours under a 12-hour light and 12-hour dark cycle. n = 6 per genotype. Two-way ANOVA shows that the two curves are not significantly different. (B) Number of sleep bouts per duration bin, before (left) and after (right) a light pulse (LP) at ZT22. n = 6 per genotype, no differences are observed. (C) Amount of sleep in % in the different sleep bout bins. Before the light pulse (LP) (left) a significant difference can be seen in the sleep bouts >128s and >512s between the two genotypes. This difference vanishes after the light pulse (right panel). n = 6, t-test, *p < 0.05. (TIF) [file pgen.1009625.s005.tif]
