# Supplementary material for: Light affects behavioral despair involving the clock gene Period 1
Source: PLoS Genet. 2021 Jul 8;17(7):e1009625. doi: 10.1371/journal.pgen.1009625 (PMC8266116; doi:10.1371/journal.pgen.1009625)
Supplement: Attachment — Submitted filename: Reply to reviewers.docx [file pgen.1009625.s016.docx]

Reviewer #1: In this manuscript, the authors found that light pulses at ZT22 change behaviors that are usually releated to depression- and anxiety. Furthermore, they found that a light pulse given at this time activates the expression of the circadian clock gene Period1 (Per1) in the lateral habenula (LHb). A total knockout and a knockdown of PER1 in the LHb leads to depression-like behavior. Therefore, the authors conclude that Per1 in the LHb might play an important role in positive effects of light therapies in depression. Additionally, the authors show that a light pulse during the second half of the dark phase causes profound changes of gene expression in the suprachiasmatic nucleus, the LHb and the nucleus accumbens (NAc) in WT mice, which were different in Per1-/- mice.

This is an interesting study of high relevance, which aims to link clinical treatment strategy to molecular mechanism. The manuscript is well-written and the figures are clearly presented. All methods the authors used are standard. However, the interpretation of the results is not very conclusive and there are some major and minor concerns that need to be considered before publication.

Major:
1) Since the authors use untreated WT mice, it might not be appropriate to address their levels of immobility in the FST test as signs of despair or depression, unless there is reason to believe that despair and depression are default traits of mice. Instead of measuring immobility, which can occur due to many reasons, it might be more meaningful to test susceptibility to develop depression-like behavior in response to stress.

For mice, swimming is a form of stress. It is of course difficult to separate a stress behavior and despair. However, if mice are under stress, like here in the forced swim test, then immobility becomes a measure of despair. The acute effects of stress are, however, not necessarily related to despair, therefore the first day is discarded in the measurements. This has been described in the original publications by Porsolt (Porsolt et al., 1977, Nature 266, 730-732; Porsolt et al., 1978, Eur. J. Phramacol, 47, 379-391). The time spent immobile is an index of depressive-like behavior and this time can be reduced by treatment with antidepressant. Therefore, the forced swim test is used as a proxy for depression. It is correct that we have not used antidepressants to modulate the behavior but instead use light as treatment. We report in this manuscript that light has similar effects in the FST as antidepressants have and that this is involving Per1 in the habenula.

2) In this context, it is also not clear whether the light pulse at ZT22 really changes depression-like behavior. It is also possible that due to the light pulse, the mice are simply more awake (and therefore more mobile) when tested a few hours later.

Mice are nocturnal, and active in the dark phase. If just activity between wild-type and Per1 animals would be different, then this should already show without a light pulse. However, this is not the case and the general activity in wild-type animals and Per1 animals is identical (Zheng et al., 2001). To illustrate this better, we have performed a sleep analysis comparing wild-type and Per1 mice. We find that the hourly percentage of sleep is identical between the two strains (suppl. Fig. S5A). The number of sleep bouts is in both genotypes not different, before and after the light pulse (Fig. S5B). Interestingly, we observe that the amount of sleep in % in function of the sleep bout duration is different between the two genotypes before the light pulse, but is identical after the light pulse (Fig. S5C). This indicates that percentage of sleep in function of sleep bout distribution is not different after a light pulse between the two genotypes but, it illustrates that the animals still can perceive light and that our observations in terms of immobility in the forced swim test are not due to lack of light perception or difference in awake state.

3) The interpretation that Per1 in the LHb mediates positive behavioral effects of light pulses on mood cannot be based on the presented data. To come to this conclusion, the authors would need to test behavior after a light pulse in ShPer1 mice.

This is of course a good point. In the knock-down model used we noticed that the control vector was interfering with light signaling. Therefore, we turned to another animal model using our newly developed floxed Per1 mice. We replaced Fig. 3 with the new animal model. Deletion of Per1 in the neurons of the LHb by injecting an AAV expressing Cre recombinase, we observe that a light pulse in these animals has no effect on immobility in the FST, much in contrast to control animals. This experiment revealed that Per1 in neurons of the LHb are important to mediate the light effect in the FST. However, Per1 in the neurons of the LHb are not responsible for the increased immobility in the FST as observed in total Per1 KO animals, suggesting that Per1 in several brain regions modulate immobility in the FST.

4) The presented RNA-based analyses are rather descriptive and the interpretation are rather speculative (e.g. the role of Per1 in the suppression of inhibitors in the NAc and the connection between genes involved in the olfactory sense and the olfactory bulb in depression).

We agree that the RNA-seq data are descriptive (as most such data are) and we do not provide an effector mechanism for Per1 downstream genes. This will also be difficult, because multiple pathways will be affected at the same time and even the snapshot we provide here will not reflect all the changes that indeed occur in this dynamic process. We have stated this in the discussion and we acknowledge that our interpretation is speculative.

Minor:
a) Fig 3C shows immobility of shPer1 mice while Fig 1C shows immobility of total Per1-/- mice. It would be much more intuitive to see how much of the effects were contributed by loss of Per1 in LHb if results of WT, Per1-/- and shPer1 mice were shown and analyzed together.

We have done this now in the new Figure 3 with the new floxed Per1 mouse model.

b) It might make sense if the authors could also show PER1 protein level of the LHb of ShPer1 mice especially when they found the knock-down of Per1 did not supress all Per1 mRNA.

We have done that now for the new mouse model.

c) In Fig 4A, the differences of the western blot bands are not clearly visible.

That is why we quantified the blots from three independent experiments. See also Yan and Silver, 2004.

d) The conclusion that Per1 is involved in the perception of reward is not in line with the findings of this study, as no change in the sucrose preference was detected.

We corrected this as suggested.

e) In line 219, the word “we” is missing.

Corrected

Reviewer #2: The authors explore the contribution of Per1 to the anti-depressant/anxiolytic effect of light and probe specific brains for their involvement. The authors found that a light pulse at ZT22 but not at ZT14 reduced time spend immobile in the FST when performed at ZT6 but not at ZT18 and that Per1 null mice were more immobile at any timepoint independent of light. Elevated O-maze mirrored this result with Per null spending less time spend in the open.
The authors found evidence for Per1 rhythms in the LHb and further found that a ZT22 light pulse upregulated Per1 expression in the lateral/medial Hb. Induction was also found in the VTA and Nacc but to a smaller extend.
Next the authors knocked down Per1 in the LHb and found increased immobility in the FST. Notably the authors also found elevated PER1 in the LHb 10hr (at ZT8) post light pulse. Finally, the authors conducted RNA Seq on brains extracted 10hrs post +/- light pulse at ZT22 and found strong diff gen expression in the Nacc in WT while this was attenuated in Per1 nulls while SCN and LHb showed profound increase in diff express genes in Per nulls. Interestingly, the VTA did not show much change in either.

This is an interesting manuscript adding to the emerging importance of the habenula and its circadian properties in mood regulation. The experiments are carefully controlled pointing to a role of the LHb local clock or local Per1 gene expression in mood regulation. Specifically intriguing is the robust RNA Seq result indicating that Per1 either in the SCN and/or the habenula suppresses pathways that in turn suppress NAc ‘activation’ by light which might be key to the antidepressant effect of light. Removal of Per1 therefore consequently leads to Nac ‘inactivation’. This finding is very compelling as the Nacc is a key player in mood regulation and the (perhaps exclusive) target of psychostimulant action.

There are a few aspects of the data presentation and discussion that need work.

The fact that Per1 is a core clock component and at the same time light inducible should be related to the findings, how can the findings be explained by an effect on the clock (phase advance…)? Is there room for an explanation based on light induction only, on pathways independent of the clock? Could it be a mix of both?

With the new animal model, we show that Per1 in the LHb is responsible for the light effects on the behavior in the FST. However, Per1 in the LHb has no effect on the base line behavior, these animals show no increased immobility in the FST in contrast to the total Per1 KO animals. Therefore, we can conclude, that Per1 in the LHb only affects the light signal and other brain regions expressing Per1 contribute to the base line behavior in the FST. Taken together it is unlikely that phase advances via the clock make the trick, since the LHb Per1 KO animals still have Per1 expression in the SCN, which drives clock phase shifting. If the clock in the SCN would mediate the process, then LHb Per1 KO animals would still respond to a light pulse in the FST, which is not what we observe.

The result in Fig4A needs more interpretation: could the increase in PER1 levels at ZT 8 suggest an advance of the PER peak meaning that it is a consequence of phase shift rather than a ‘plastic’ long-term change?
In this regard the long lasting effect of a single light pulse on FST (5days!) is hard to fathom…what could possibly explain such sustained effect?

We agree with the reviewer that the long term effects can not be understood with the one time point analysis we did here. The main message of the manuscript is that light therapy works in mice and that Per1 in the LHb is invovled in this process. The RNAseq data only illustrates early events of this process and the long-term effects need deeper analysis with additional time points. We describe this in the discussion.

RE RNA Seq, the volcano plots clearly show changes for both up and down regulated genes, this needs to be more carefully addressed (e.g the term ‘induction’ is incorrect because many genes are becoming suppressed), perhaps use ‘diff regulated’.

We changed this accordingly

The lack of response in the VTA is interesting, could it be that the SCN/LHb acts on the Nacc without the VTA as mediator? The Nacc is innervated by many regions not only the VTA. The current explanation given for the lack of response to light of the VTA transcriptome is not clear/too hand-wavy to me.

We did a one time point analysis at ZT8 to illustrate that the light pulse experiment worked. With this we can not explain dynamic processes that are occuring here. We state this in the discussion. The RNAseq experiment, however, shows spacial changes at one time point but not temporal changes, which would be another question to address in the future. This is mentioned in the discussion.

The posture result in Fig.3 is very significant, perhaps repeat in global Per-/- and add to figure 1? Not critical but would strengthen the knock down approach!

We deleted the knock-down approach and replaced it with the local conditional knock-out model. The new model shows that Per1 in the LHb does not affect despair nor anxiety, but only the light effect on despair.
