# Supplementary material for: Light affects behavioral despair involving the clock gene Period 1
Source: PLoS Genet. 2021 Jul 8;17(7):e1009625. doi: 10.1371/journal.pgen.1009625 (PMC8266116; doi:10.1371/journal.pgen.1009625)
Supplement: Attachment — Submitted filename: Response to Reviewers.docx [file pgen.1009625.s017.docx]

**Response to Reviewers:**

Reviewer #1: I am sorry to hear that the authors had problems with their control vector. I give them all the more credit for the extensive work they did to answer the questions from us reviewers. The paper is much improved and I don't see any other issues that need to be worked on.

We thank the reviewer for his time to evaluate our manuscript.

Reviewer #2: In this revision, the authors replaced a key element of the paper, the shRNAi knock down of Per1 in the LHb, with a Cre loxP approach, and explain this with an issue with the control vector under conditions of light induction. It is not clear why they removed all of the shRNAi results, as for instance the increased FST immobility of the knock down mice was a result independent of light, where the empty/scrambled vector had no effect but the shRNAi construct did. If there is no concern with the data, the authors should retain this shRNAi result (increased immobility).

We have changed our approach due to the fact that the light treatment did not work in the control shRNA injected animals, probably due to effects of the overexpression of GFP in this approach. Hence, lack of an effect of light treatment on the Per1 knock-down animals can not be proven. This indicated that the shRNA approach is not suitable. That was the reason to remove all shRNA data. We are confident that the novel approach is reliable.

Regarding the new model, demonstration of selective disruption (the authors should use such term and not ‘knock down’ in the context of Cre loxP) is not convincing. The IHC in Fig. 3 appears rather unspecific. Shouldn’t PER1 be predominantly nuclear? There seem to be no hint of any nuclear staining.

We performed additional IHC experiments (Fig. 3C). A magnification to show nuclear localization of PER1 is shown below (blue = DAPI stained nuclei, pink = PER1). The magnification is from Fig. 3C.

Has the antibody been validated?

Yes, see Schnell et al., 2015

How does it look in a WT versus Per1-/- SCN? The authors need to show higher magnification images preferably co-labeled with a neuronal marker to demonstrate that the IHC is specific (side-by-side with Per1-/- sections). Alternatively the authors may want to employ FISH (RNAscope) to demonstrate efficacy of the disruption. A count of all PER1+ cells (co-labeled with a neuronal maker) in the ROI (=LHb) of selective disrupted and WT mice would be important to quantify the efficacy. Also, a lower mag image would be helpful to demonstrate the spatial specificity of Cre action. The IHCs of the AAV-GFPs in Fig. S3A indicate that even AAV6 seems only to affect half of the LHb…so a careful assessment of disruption efficacy is very much needed.

We have performed additional IHC experiments and show the requested controls now in Fig. 3C.

The authors still fail to acknowledge that light does not simply induce transcripts but affects the transcriptomes fairly evenly in both directions for all tissues and genotypes, if one uses the volcano plots as guides.

This is correct and we acknowledge this now in the text.

In this context, the identification of differentially expressed transcripts needs explanation. The cut off p-values below which a transcriptional change is considered significant seems to vary between tissues and genotypes according the black labeled dots in the volcano plots, this needs clarification.

1. The RNAseq data from each brain region was analyzed independently, meaning that each gene, the fold change and statistical tests only apply for the comparisons within a particular brain region.

2. All the analyses in the DESeq2 program used the same calculation settings and selection criteria of differentially expressed genes. In detail, the following procedures were the same for all the brain regions.

3. In the DESeq2 program, genes with **too low raw counts** were removed before the calculation of fold change and statistical tests.   
**A gene with too low raw counts** is defined as a gene where the sum of the raw counts in all the samples is smaller than 25 counts.

4. After the calculation of fold change and statistical tests, genes with p-value assigned as NA (Not available) were removed.
The DESeq2 program assigns p-value as NA to a gene when a gene contains count outliers.

5. The DESeq2 program performed the Wald's test as the default statistical test option to generate the p-value. In addition to the Wald's test, the DESeq program also performs the Benjamini-Hochberg (BH) method to generate padj value (p adjusted), to account for false positives.

If the null hypothesis of the Wald's test is true, i.e. there is no difference in the gene expression level, by the definition of p-value as < 0.05, there would still be 5% of the genes having a p-value < 0.05. We observed about 18,000 - 20,000 genes expressed in the samples, 5% of the total expressed genes could have a p-value < 0.05,  meaning that 900 - 1000 genes could be false positive if there is no difference between the conditions we were comparing. Therefore, p-value < 0.05 is not used as a cut-off for selecting significantly differentially expressed genes.

In this paper, **significant genes** are defined as genes having the padj value smaller than 0.1, meaning that our acceptable false discovery rate (FDR) is 10%.

For the details of the statistical methods in DESeq2, please refer to the paper Love et al. Genome Biology (2014) 15:550 and the DESeq2 package documentation available at [bioconductor.org](http://bioconductor.org/) (<https://bioconductor.org/packages/release/bioc/html/DESeq2.html>)

6. The y-axis of the volcano plot is -log10(p-value) not the padj. Since the adjustment procedure creates a slightly different cut-off for the data-set of each brain region, the corresponding ‘cut-off’ for the p-value is slightly different in all the panels (which also explains that the cut-off significant to non-significant is roughly a line).

7. The documentation, programming scripts, all the input and output data files of the whole bioinformatic analysis part for this study can be found in the following github repository link. However, it is in the private mode right now. It will be in the public mode once the manuscript is accepted.

<https://github.com/kayihui/effect_of_light_on_brain_regions>

Optional point:
There are advantages and disadvantages of any technique in experimentation, the RNAseq experiment, differentially gene expression analysis and the TopGO analysis provide an birds eye view on the changes at the genome-wide level, which also serve as an exploration on what genes or pathways might be of interest to be studied further.  The transcriptome data itself is only a snapshot of what happened at a specific time point. The data only suggested Per1-dependent and Per1-independent light-induced gene expression changes in the various brain regions. However, it would not be able to answer complex questions on specific biological mechanisms or direct causation. The role and possible mode of action of Per1 in the observed phenotype is supported by other evidence in the paper, rather than the RNASeq analysis alone.

With regard to the NAc the authors use the term induction of transcript thereby giving the impression as if light leads to ‘activation’ of the Nacc 10 hrs later. However, the volcano plot does not allow for such interpretation as induced and suppressed transcript seem to roughly equal in number (the suppressed transcripts seem to be even higher, gauging form the volcano plots).

This is correct and we adapted the text accordingly.

Because of this, the notion that Per1 acts as a suppressor is not supported by the transcriptomics data, rather Per1’s role in the LHb is to prevent ANY action of light on the LHb transcriptome which would otherwise lead to negative action onto despair circuits. Given that Per1Ko prevents even downregulation of transcripts, Per1 might have a higher order function here e.g. preventing light from accessing any aspect of transcriptional regulation.

This is correct, because PER1 can suppress inhibitors and this may result in activation of genes if PER1 is lacking. Therefore, we do not state anymore the direction of change in gene expression.

In general I am not convinced that one should engage in interpretation of the RNAseq data with regard to depression-related behaviors and circuits/regions without a detailed look at individual transcripts (and perhaps more time points), especially given the fact that light seem to suppress as many transcripts as it is activating. At this point it seem unclear what the transcriptional changes actually mean: activation or silencing of the circuits? Or none of the above? The pathway analysis provided is really very coarse and clearly incomplete as it does not address at all the numerous downregulated transcripts in the Nacc and elsewhere.

We agree with the reviewer. This is why we only make a general statement on the pathways that may be affected. Interstingly, however, data on depression related to smell and the olfactory bulb support the general statement we make.

Given that the LHb/Habenula is considered one of the most rhythmic extra-SCN brain oscillators (see e.g. Guilding Piggins et al. 2007), the authors should discuss a possible contribution of Per1 through the local LHb clock.

Lack of Per1 in the LHb most likely will shorten the period of the LHb oscillator. But this oscillator most likely plays no role in the manifestation of increased immobility in the FST in the absence of a light pulse (Fig. 3F). Only lack of Per1 in additional brian regions and tissues (total KO) show increased immobility in the FST without a light pulse (Fig. 1C). We adapted the text accordingly (lines 322-327).

Finally, the correlation/reference to SAD is perhaps overreaching, given that mice are nocturnal and thus much of the effects of light on mood might be actually inverse.

Clock gene expression and Per gene induction are identical in nocturnal and diurnal animals. Even the resetting responses are the same (Mahoney, M., et al. J. Biol. Rhythms 16, 149–162, 2001). Furthermore, our data here show a striking parallel of the efficiency of light treatment to the human protocol (Wirz-Justice et al., ref 11 in the paper). It is correct that some clock regulated processes can be opposite, but here we show that this is probably not the case for mood related behaviors.

Evidence for this is provided by light masking, which is completely inverse and can be viewed as inverse arousal regulation. And arousal is relevant, as one of the hall marks of SAD is sleep problems, specifically difficulty to fall asleep during periods of winter gloom. Also, light therapy improves mood during the daytime, however, the ZT22 light pulse (2hours before ‘wake up’) has no effect on the ‘daytime’ mood of mice (ZT18), ZT6 is really a mid-sleep time point.

The light pulse in the early morning in SAD treatment is most efficient in the dark phase (ZT22) in humans. The later the pulse occurs the less efficient the treament in humans (Wirz-Justice et al., ref 11 in the paper). The beneficial effect on mood is in humans as well as in mice is in the light phase. It is correct that for humans this is the activity phase and in mice the rest phase.

In this context, Huang et al. (Neuron 2018) did apply ‘light therapy’ during the daytime (ZT1-3) and over a course of 10/14days. Regardless, using a nocturnal model for SAD is clearly a challenge and that needs to be acknowledged more in the discussion.

Interestingly, a recent paper failed to reproduce this effect of light at ZT1-3 and it has several common authors to Huang et al.! (Fig. 1, An et al., Nat. Neurosci., 23, 869-880), 2020. The An et al. paper applied light pulses at ZT13-15. They observe a depression inducing effect of light at that time (early night). Hence, there is probably a depression response curve (comparable to the phase response curve) with depression inducing effects of light in the early night (Z13-15) and a depression reducing effetc of light in the late night (ZT22).

Minor:
-The abstract does not take into account the new model, selective disruption of Per1 in the LHb.

We thank the reviewer for this comment and changed the abstract accordingly.

-“At this time point, light not only causes eventual adaptation of the mammalian circadian clock to a new time zone but is also most effective in the treatment of some forms of depression, such as seasonal affective disorder [11].”
Is this true, has light at ZT22 (=2hrs before light on i.e. before wake up) been used for SAD treatment?

Yes, see (Wirz-Justice et al., ref 11 in the paper)

**Have all data underlying the figures and results presented in the manuscript been provided?**
Large-scale datasets should be made available via a public repository as described in the *PLOS Genetics* [data availability policy](http://journals.plos.org/plosgenetics/s/data-availability), and numerical data that underlies graphs or summary statistics should be provided in spreadsheet form as supporting information.

Reviewer #1: Yes

Reviewer #2: Yes

PLOS authors have the option to publish the peer review history of their article ([what does this mean?](https://journals.plos.org/plosgenetics/s/editorial-and-peer-review-process#loc-peer-review-history)). If published, this will include your full peer review and any attached files.


If you choose “no”, your identity will remain anonymous but your review may still be made public.


**Do you want your identity to be public for this peer review?** For information about this choice, including consent withdrawal, please see our [Privacy Policy](https://www.plos.org/privacy-policy).

Reviewer #1: No

Reviewer #2: No
