# Supplementary material for: Light affects behavioral despair involving the clock gene Period 1
Source: PLoS Genet. 2021 Jul 8;17(7):e1009625. doi: 10.1371/journal.pgen.1009625 (PMC8266116; doi:10.1371/journal.pgen.1009625)
Supplement: Attachment — Submitted filename: Responses to Reviewers Questions3.docx [file pgen.1009625.s018.docx]

Responses to Reviewers Questions

Reviewer #2: The authors addressed all comments to my satisfaction, except for one:

My point:
Evidence for this is provided by light masking, which is completely inverse and can be viewed as inverse arousal regulation. And arousal is relevant, as one of the hall marks of SAD is sleep problems, specifically difficulty to fall asleep during periods of winter gloom. Also, light therapy improves mood during the daytime, however, the ZT22 light pulse (2hours before ‘wake up’) has no effect on the ‘daytime’ mood of mice (ZT18), ZT6 is really a midsleep time point.

And the authors response:
The light pulse in the early morning in SAD treatment is most efficient in the dark phase (ZT22) in humans. The later the pulse occurs the less efficient the treament in humans (Wirz-Justice et al., ref 11 in the paper). The beneficial effect on mood is in humans as well as in mice is in the light phase. It is correct that for humans this is the activity phase and in mice the rest phase.

My comment:
The authors really need to explain why an improvement at ZT6 is relevant and speaks to the human situation as it would equate to waking up a human in the middle of the night to conduct the behavioral test, which is not something that is typically done in humans.

Yes, this is correct. However, the sleep patterns of humans and mice is different. Mice have a more fragmented sleep compared to humans.

Thus the correct time for testing would seem to be the wake period (ZT12-ZT24), not ZT6.
The authors should discuss this limitation as it speaks also more broadly to the meaningfulness of behavior testing in nocturnal rodents during day time to learn something relevant for the human situation.
Also with regard to the same clock phasing in nocturnal and diurnal animals: this is true for the SCN but it seems that the output is quite immediately converted, suggesting that the arousal rhythmicity that spreads across the brain is really very much antiphasic in such species.

Yes, this is of course correct if the observations we make are clock dependent. Here we do not know whether this is indeed the case, and hence this is a limitation for the interpretation of the data. We mention this now in the discussion.

Line 285: Of note is that the testing of mice in the FST was in their rest phase, whereas in humans it is in the activity phase. This puts a limitation on our interpretations.
